# Supplementary material for: Why is the labor epidural rate low and cesarean delivery rate high? A survey of Chinese perinatal care providers
Source: PLoS One. 2021 May 21;16(5):e0251345. doi: 10.1371/journal.pone.0251345 (PMC8139447; doi:10.1371/journal.pone.0251345)
Supplement: S1 Appendix — (PDF) [file pone.0251345.s001.pdf]

S1. Appendix: The survey questions in both the original language (Chinese) and English

Demographic data related questions:

1. 您的职业是？（单选题）What is your medical speciality? (multiple choice)
    a. 产科医生 Obstetrician
    b. 麻醉科医生 Anesthesiologist
    c. 助产士 Midwife
    d. 产科护士 Labor & delivery nurse
    e. 医院行政人员 Hospital administrator
    f. 其他，请注明 Others (Open)
2. 贵医院所在的省份是？（单选题）

    Which area is your hospital located? (multiple choice)

    （31个大陆直辖市、省、自治区)

    (A list of all 31 Province/Autonomous region/Metropolis in Mainland China）
3. 贵医院的单位性质是？（单选题）

    What type of hospital do you work? (multiple choice)
    a. 综合医院 General hospital
    b. 妇产专科医院 Women's hospital
    c. 民营医院 Private hospital
    d. 其他，请注明（开放选项）：Others (Open)
4. 贵医院的评级？（单选题）What level of hospital do you work? (multiple choice)
    a. 三级以上（包含三级）

        ≥ Level III (tertiary hospital)
    b. 二级以上（包含二级），三级以下（不包含三级）

        ≥ Level II (municipal hospital) ~ < Level III (tertiary hospital)

c. 二级以下（不包含二级）

< Level II (municipal hospital)

<u>Survey questions</u>：

1. 您认为造成中国高剖宫产率的原因可能包括哪些？（请勾画您认为适合的所有答案，可以多于一个）

   What would be the reasons for the high cesarean rate in China in your opinion (≥ one choice)?

   a. 我们知道剖宫产并发症危害产妇，但病人要求做，因为怕医闹，我们不敢不做

      Try to avoid lawsuit or yiinao/yibao† due to parturient request even knowing cesarean delivery is harmful without medical indications

   b. 我们知道剖宫产并发症危害产妇，但病人要孩子在“良辰吉日”出生，这是中国文化，我们不能不满足病人要求

      Chinese superstition culture for selecting particular date of their childbirths even knowing cesarean delivery is harmful without medical indications

   c. 剖宫产很安全，不会对婴儿造成危害

      Cesarean delivery is very safe and not harmful to babies

   d. 剖宫产很安全，不会对母亲造成危害

      Cesarean delivery is very safe and not harmful to mothers

   e. 剖宫产很安全，不会给母亲留后遗症

      Cesarean delivery is very safe and results in no long-term complications

   f. 剖宫产很安全，而且比做硬膜外收入高

      Cesarean delivery is very safe along with better incentive than vaginal delivery with labor epidural analgesia

   g. 其他，请注明（开放选项）：

      Others (open)

2. 您认为硬膜外分娩镇痛使用率不高的原因是什么？（请勾画您认为适合的所有答案，可以多于一个）

   What reasons do you think cause a low labor epidural analgesia rate (≥ one choice)?

   a. 剖宫产比硬膜外安全，硬膜外中的局麻药和阿片类药物对胎儿有害

      Cesarean delivery is safer than vaginal delivery because local anesthetics and opioid in labor epidural analgesia are bad for fetus

   b. 硬膜外后，产妇并发症多

      More complications from vaginal delivery with labor epidural analgesia than cesarean delivery

   c. 剖宫产比硬膜外挣钱多，硬膜外会减少科室收入

      More incentive from cesarean delivery and lose money if providing labor epidural analgesia for vaginal delivery

   d. 我们从来没做过硬膜外分娩镇痛，不知道如何做

      Never have experiences of labor epidural analgesia and do not know how to do it

   e. 我们以前做过硬膜外镇痛，但效果不好，就放弃了

      Gave it up because of its ineffectiveness from previous experiences

   f. 麻醉医生人员不足，根本没有时间做硬膜外

      Lack of anesthesia manpower

   g. 助产士太忙，没有时间观察硬膜外分娩镇痛的病人

      Lack of midwife or nurse manpower to monitor patients

   h. 产科医生不愿意做

      Obstetricians do not want to provide labor epidural analgesia service

   i. 医院领导不愿意做

      Hospital administrators do not want to provide labor epidural analgesia service

   j. 麻醉科医生不愿意做

Anesthesiologists do not want to provide labor epidural analgesia service

k. 产妇不愿意

Parturients do not want labor epidural analgesia

l. 产妇家属不同意

Family members do not want labor epidural analgesia

m. 其他原因，请注明（开放选项）：

Others (open)

3. 医院领导支持很重要，您觉得领导应该怎样支持无痛分娩？（请勾画您认为适合的所有答案，可以多于一个）

What to suggest hospital administrators to promote labor epidural analgesia service presuming they are willing to support (≥ one choice)?

a. 增加麻醉科医生

Increase in anesthesia manpower

b. 增加奖金

Increase in incentive amount

c. 增加助产士

Increase in midwife manpower

d. 增加产科医生

Increase in obstetricians

e. 增加产科护士

Increase in labor & delivery nurses

f. 其他举措，请注明（开放选项）：

Others (open)
