# Supplementary material for: Why is the labor epidural rate low and cesarean delivery rate high? A survey of Chinese perinatal care providers
Source: PLoS One. 2021 May 21;16(5):e0251345. doi: 10.1371/journal.pone.0251345 (PMC8139447; doi:10.1371/journal.pone.0251345)
Supplement: Attachment — Submitted filename: PlosOne.pdf [file pone.0251345.s003.pdf]

Dear Editor,

I am honored to be invited to review this article.

As an obstetrician for more than 16 years, I do realize that cesarean delivery rates overall are very different. The differences in rates depend on not only medical indications but also social-economic status. We do have a lot of clinical studies addressing unnecessary cesarean deliveries medically. Unfortunately, the vast majority of cesarean deliveries occur far beyond our majorities of research. It is fortunate that the authors picked up this topic in this very unique view. The evidence that the authors provided have convinced me that providing epidural labor analgesia can safely reduce the cesarean delivery rate. This conclusion was drawn not only from their own large impact studies in different levels of hospitals but also from RCT studies reviewed by the Cochrane group in 2018. In other words, the background is solid and real.

As I read the manuscript, I also realized that the unnecessary cesarean deliveries in China have equaled the total amount of cesarean deliveries annually in the United States. I felt that this topic and approach would also be very beneficial to the medical societies in the rest of the world other than China. The high rates of cesarean delivery are epidemic if not pandemic epidemiologically since the unnecessary cesarean deliveries have clearly causal effects on maternal and infant morbidity and mortality. There are some unique cultural aspects, such as superstitious culture which was only applied to the local community. However, such a situation occurs in about 20% of the world population and maybe even more in other Asian countries around.

Having talked about many positive aspects of this manuscript, I would like to make a few comments and recommendations:

Major issues:

1) Table 1 and Table 2 could be presented better. The authors may use histogram for better and more clear view.
2) Web-based survey is a relatively new form with a lower respondent rate than traditional surveys via email, mail, or interview. The authors may analyze distribution bias, and discuss related biases to help readers to interpret data objectively.

Minimal issues:

1) If the authors keep Table 1 and Table 2, two columns for each specialty should become one in the format “xxx (%)” in order to simplify the tables..
2) It may be better to list the survey questions as an appendix at the end.

I am so impressed by the feasible solution concluded in the study. I am very glad that you accepted this manuscript and started its reviewing process.
