# Supplementary material for: Why is the labor epidural rate low and cesarean delivery rate high? A survey of Chinese perinatal care providers
Source: PLoS One. 2021 May 21;16(5):e0251345. doi: 10.1371/journal.pone.0251345 (PMC8139447; doi:10.1371/journal.pone.0251345)
Supplement: Attachment — Submitted filename: Response to Reviewers.docx [file pone.0251345.s004.docx]

RE: PONE-D-21-00405

Why is the labor epidural rate low and cesarean delivery rate high? A survey of Chinese perinatal care providers

PLOS ONE

Dear Dr. Wei,

Thank you for your constructive comments and suggestions. The following is our response to your comments and reviewers’.

Thank you for your time and efforts.

Sincerely,

Ling-Qun Hu, MD

The corresponding author

=======================================

Dear Dr. Hu,

Thank you for submitting your manuscript to PLOS ONE. After careful consideration, we feel that it has merit but does not fully meet PLOS ONE’s publication criteria as it currently stands. Therefore, we invite you to submit a revised version of the manuscript that addresses the points raised during the review process.

Please submit your revised manuscript by Mar 19 2021 11:59PM. If you will need more time than this to complete your revisions, please reply to this message or contact the journal office at plosone@plos.org. When you're ready to submit your revision, log on to <https://www.editorialmanager.com/pone/> and select the 'Submissions Needing Revision' folder to locate your manuscript file.

Please include the following items when submitting your revised manuscript:

- A rebuttal letter that responds to each point raised by the academic editor and reviewer(s). You should upload this letter as a separate file labeled 'Response to Reviewers'.
- A marked-up copy of your manuscript that highlights changes made to the original version. You should upload this as a separate file labeled 'Revised Manuscript with Track Changes'.
- An unmarked version of your revised paper without tracked changes. You should upload this as a separate file labeled 'Manuscript'.

If you would like to make changes to your financial disclosure, please include your updated statement in your cover letter. Guidelines for resubmitting your figure files are available below the reviewer comments at the end of this letter.

If applicable, we recommend that you deposit your laboratory protocols in [protocols.io](http://protocols.io/) to enhance the reproducibility of your results. Protocols.io assigns your protocol its own identifier (DOI) so that it can be cited independently in the future. For instructions see: <http://journals.plos.org/plosone/s/submission-guidelines#loc-laboratory-protocols>

We look forward to receiving your revised manuscript.

Kind regards,

Huafeng Wei, MD, PhD

Academic Editor

PLOS ONE

Journal requirements:

When submitting your revision, we need you to address these additional requirements.

1. Please ensure that your manuscript meets PLOS ONE's style requirements, including those for file naming. The PLOS ONE style templates can be found at

<https://journals.plos.org/plosone/s/file?id=wjVg/PLOSOne_formatting_sample_main_body.pdf> and

<https://journals.plos.org/plosone/s/file?id=ba62/PLOSOne_formatting_sample_title_authors_affiliations.pdf>

A: Thank you for the templates and the format for both the main body and the authors’ information are adapted accordingly

2. Please include additional information regarding the survey or questionnaire used in the study and ensure that you have provided sufficient details that others could replicate the analyses. For instance, if you developed a questionnaire as part of this study and it is not under a copyright more restrictive than CC-BY, please include a copy, in both the original language and English, as Supporting Information. If the original language is written in non-Latin characters, for example Amharic, Chinese, or Korean, please use a file format that ensures these characters are visible.

A: Thank you. We have added the list of questions as SI Appendix in both Chinese (the original language for the survey) and English. Because they were written in Chinese, a separate file is uploaded as requested. The questions were generated by our authors without restricted protection by neither trademark nor copyright.

3. In your Methods section, please include additional information about your dataset and ensure that you have included a statement specifying whether the collection method complied with the terms and conditions for the application WeChat.

A: Other than geological data for geological analysis and IP address for restricting one respondent from providing multiple answers, other detailed personal information was not requested and collected. WeChat was the main distribution platform in addition to other two professional websites mentioned in the text. The WeChat ID or other log-in information was not requested for the survey and then unable to be presented as a part of the survey results. We have added a sentence to make it clearer to the readers: “The questionnaire was created using https://www.wenjuan.com/s/zMRbUn4/ and was promoted in multiple professional groups run by Jicheng Anesthesia Network (http://www.jcmzw.com) and Chinese Obstetrics and Gynecology Network (http://www.obgy.cn/) (for obstetricians, gynecologists and midwives) via WeChat (the most widely used social media used by Chinese medical professionals to share knowledge and discuss clinical questions) and their official web sites. All surveys were completed based on voluntary participation without any incentives and required logging-in information .”

4. Please state whether you validated the questionnaire prior to on study participants. Please provide details regarding the validation group within the methods section.

A: All the questions have been discussed prior to the survey among our leaders of NPLD-GHI for their clarity, objectivity and feasibility. The language has been carefully examined by the author Z Cai, who is one of the key academic editors for “Journal of No Pain Labor & Delivery - Global Health Initiative'', an obstetrician, the chair of the Obstetrics Department in Aviation General Hospital, and associate professor in China Medical University. An anonymous online survey questionnaire written in Chinese asked for respondent occupation, locations of work, opinions about LEA and its complications, their concerns,, medical compensation, reasons for poor utilization, and suggestions for improvement. The questionnaire was initiated by the first author “Zhao, P”, whose first language is Chinese, and confirmed by the author “Cai, Z”, an obstetrician-in-chief, for both linguistic and professional wording. The final set of the survey questions attached as an appendix (S1 Appendix) was validated by Chinese-American healthcare providers from Northwestern University, Harvard Medical School, the Ohio State University, New York University, Howard Medical University, Georgetown Washington University, Mount Sinai at New York, Stanford University, Duke University, and some Chinese institutions. ”

5. Please include your tables as part of your main manuscript and remove the individual files. Please note that supplementary tables (should remain/ be uploaded) as separate "supporting information" files.

A: Thank you for pointing out the specific requirement for tables. Our updated version has converted the tables to Fig 2 and Fig 3 for better presentation. We have added Fig 2 and 3 titles and their legends in appreciated sections in the main text and uploaded them as required. So did Fig 1 as required as well.

6. Thank you for stating the following in the Competing Interests section:

"The authors have declared that no competing interests exist."

We note that one or more of the authors are employed by a commercial company: ApgarCARE International, Inc.

(1) Please provide an amended Funding Statement declaring this commercial affiliation, as well as a statement regarding the Role of Funders in your study. If the funding organization did not play a role in the study design, data collection and analysis, decision to publish, or preparation of the manuscript and only provided financial support in the form of authors' salaries and/or research materials, please review your statements relating to the author contributions, and ensure you have specifically and accurately indicated the role(s) that these authors had in your study. You can update author roles in the Author Contributions section of the online submission form.

Please also include the following statement within your amended Funding Statement.

“The funder provided support in the form of salaries for authors [insert relevant initials], but did not have any additional role in the study design, data collection and analysis, decision to publish, or preparation of the manuscript. The specific roles of these authors are articulated in the ‘author contributions’ section.”

If your commercial affiliation did play a role in your study, please state and explain this role within your updated Funding Statement.

(2) Please also provide an updated Competing Interests Statement declaring this commercial affiliation along with any other relevant declarations relating to employment, consultancy, patents, products in development, or marketed products, etc.

Within your Competing Interests Statement, please confirm that this commercial affiliation does not alter your adherence to all PLOS ONE policies on sharing data and materials by including the following statement: "This does not alter our adherence to PLOS ONE policies on sharing data and materials.” (as detailed online in our guide for authors http://journals.plos.org/plosone/s/competing-interests) . If this adherence statement is not accurate and there are restrictions on sharing of data and/or materials, please state these. Please note that we cannot proceed with consideration of your article until this information has been declared.

Please include both an updated Funding Statement and Competing Interests Statement in your cover letter. We will change the online submission form on your behalf.

Please know it is PLOS ONE policy for corresponding authors to declare, on behalf of all authors, all potential competing interests for the purposes of transparency. PLOS defines a competing interest as anything that interferes with, or could reasonably be perceived as interfering with, the full and objective presentation, peer review, editorial decision-making, or publication of research or non-research articles submitted to one of the journals. Competing interests can be financial or non-financial, professional, or personal. Competing interests can arise in relationship to an organization or another person. Please follow this link to our website for more details on competing interests: <http://journals.plos.org/plosone/s/competing-interests>

A: ApgarCARE International, Inc. is a not for profit organization registered in the State of Illinois, USA, established for No Pain Labor & Delivery - Global Health Initiative program. There is neither funding support for this survey nor any of the authors receiving any stipend for the survey. All the work was volunteered without any third parties involved financially or physically. Therefore, the statement of "The authors have declared that no competing interests exist." still remains the same. To avoid any confusion, Shuwei Yang, only one of the authors, could be a volunteer of “No Pain Labor & Delivery - Global Health Initiative, ApgarCARE International, Inc,” or “No Pain Labor & Delivery - Global Health Initiative” instead, based on PLOSOne’s regulation if needed.

7. We note that Figure 1 in your submission contains map images which may be copyrighted. All PLOS content is published under the Creative Commons Attribution License (CC BY 4.0), which means that the manuscript, images, and Supporting Information files will be freely available online, and any third party is permitted to access, download, copy, distribute, and use these materials in any way, even commercially, with proper attribution. For these reasons, we cannot publish previously copyrighted maps or satellite images created using proprietary data, such as Google software (Google Maps, Street View, and Earth). For more information, see our copyright guidelines: <http://journals.plos.org/plosone/s/licenses-and-copyright>.

We require you to either (1) present written permission from the copyright holder to publish these figures specifically under the CC BY 4.0 license, or (2) remove the figures from your submission:

(1) You may seek permission from the original copyright holder of Figure 1 to publish the content specifically under the CC BY 4.0 license.

We recommend that you contact the original copyright holder with the Content Permission Form (http://journals.plos.org/plosone/s/file?id=7c09/content-permission-form.pdf) and the following text:

“I request permission for the open-access journal PLOS ONE to publish XXX under the Creative Commons Attribution License (CCAL) CC BY 4.0 (http://creativecommons.org/licenses/by/4.0/). Please be aware that this license allows unrestricted use and distribution, even commercially, by third parties. Please reply and provide explicit written permission to publish XXX under a CC BY license and complete the attached form.”

Please upload the completed Content Permission Form or other proof of granted permissions as an "Other" file with your submission.

In the figure caption of the copyrighted figure, please include the following text: “Reprinted from [ref] under a CC BY license, with permission from [name of publisher], original copyright [original copyright year].”

(2) If you are unable to obtain permission from the original copyright holder to publish these figures under the CC BY 4.0 license or if the copyright holder’s requirements are incompatible with the CC BY 4.0 license, please either i) remove the figure or ii) supply a replacement figure that complies with the CC BY 4.0 license. Please check copyright information on all replacement figures and update the figure caption with source information. If applicable, please specify in the figure caption text when a figure is similar but not identical to the original image and is therefore for illustrative purposes only.

The following resources for replacing copyrighted map figures may be helpful:

USGS National Map Viewer (public domain): http://viewer.nationalmap.gov/viewer/

The Gateway to Astronaut Photography of Earth (public domain): http://eol.jsc.nasa.gov/sseop/clickmap/

Maps at the CIA (public domain): https://www.cia.gov/library/publications/the-world-factbook/index.html and https://www.cia.gov/library/publications/cia-maps-publications/index.html

NASA Earth Observatory (public domain): http://earthobservatory.nasa.gov/

Landsat: <http://landsat.visibleearth.nasa.gov/>

USGS EROS (Earth Resources Observatory and Science (EROS) Center) (public domain): http://eros.usgs.gov/#

Natural Earth (public domain): http://www.naturalearthdata.com/

A: Thank you for reminding me of the copyright issue. We will not use the figure generated from the survey site that might be protected or restricted by CC-BY for our presentation although there is no clear statement addressing it at <https://www.wenjuan.com/register/protocol>. I believe that together to answer the reviewer 2’s comment “Major Issue #2”, a figure presenting the percentage relationship between the population and the respondents to the survey in geological locations would be a better presentation. Please find a separate file for the updated Figure 1.

Reviewers' comments:

Reviewer's Responses to Questions

**Comments to the Author**

1. Is the manuscript technically sound, and do the data support the conclusions?

The manuscript must describe a technically sound piece of scientific research with data that supports the conclusions. Experiments must have been conducted rigorously, with appropriate controls, replication, and sample sizes. The conclusions must be drawn appropriately based on the data presented.

Reviewer #1: Yes

Reviewer #2: Yes

2. Has the statistical analysis been performed appropriately and rigorously?

Reviewer #1: Yes

Reviewer #2: Yes

3. Have the authors made all data underlying the findings in their manuscript fully available?

The [PLOS Data policy](http://www.plosone.org/static/policies.action#sharing) requires authors to make all data underlying the findings described in their manuscript fully available without restriction, with rare exception (please refer to the Data Availability Statement in the manuscript PDF file). The data should be provided as part of the manuscript or its supporting information, or deposited to a public repository. For example, in addition to summary statistics, the data points behind means, medians and variance measures should be available. If there are restrictions on publicly sharing data—e.g. participant privacy or use of data from a third party—those must be specified.

Reviewer #1: Yes

Reviewer #2: Yes

4. Is the manuscript presented in an intelligible fashion and written in standard English?

PLOS ONE does not copyedit accepted manuscripts, so the language in submitted articles must be clear, correct, and unambiguous. Any typographical or grammatical errors should be corrected at revision, so please note any specific errors here.

Reviewer #1: Yes

Reviewer #2: Yes

5. Review Comments to the Author

Please use the space provided to explain your answers to the questions above. You may also include additional comments for the author, including concerns about dual publication, research ethics, or publication ethics. (Please upload your review as an attachment if it exceeds 20,000 characters)

Reviewer #1: Dear Editor,

I am honored to be invited to review this article.

As an obstetrician for more than 16 years, I do realize that cesarean delivery rates

overall are very different. The differences in rates depend on not only medical indications but

also social-economic status. We do have a lot of clinical studies addressing unnecessary

cesarean deliveries medically. Unfortunately, the vast majority of cesarean deliveries occur far

beyond our majorities of research. It is fortunate that the authors picked up this topic in this very

unique view. The evidence that the authors provided have convinced me that providing epidural

labor analgesia can safely reduce the cesarean delivery rate. This conclusion was drawn not

only from their own large impact studies in different levels of hospitals but also from RCT studies reviewed by the Cochrane group in 2018. In other words, the background is solid and real.

As I read the manuscript, I also realized that the unnecessary cesarean deliveries in

China have equaled the total amount of cesarean deliveries annually in the United States. I felt

that this topic and approach would also be very beneficial to the medical societies in the rest of

the world other than China. The high rates of cesarean delivery are epidemic if not pandemic

epidemiologically since the unnecessary cesarean deliveries have clearly causal effects on

maternal and infant morbidity and mortality. There are some unique cultural aspects, such as

superstitious culture which was only applied to the local community. However, such a situation

occurs in about 20% of the world population and maybe even more in other Asian countries

around.

A: Thank you.

Having talked about many positive aspects of this manuscript, I would like to make a few

comments and recommendations:

Major issues:

1) Table 1 and Table 2 could be presented better. The authors may use histogram for

better and more clear view.

A: Thank you and both tables have been converted into histograms labeled Fig 2 and Fig 3 uploaded in separate files.

2) Web-based survey is a relatively new form with a lower respondent rate than traditional

surveys via email, mail, or interview. The authors may analyze distribution bias, and

discuss related biases to help readers to interpret data objectively.

A: Thank you for your comment. We have created a figure to replace the previous Figure 1. The geological distribution of the respondents to the survey has been analyzed. Also, please see the answer to the editor’s comment #7 written earlier as well.

Minimal issues:

1) If the authors keep Table 1 and Table 2, two columns for each specialty should become

one in the format “xxx (%)” in order to simplify the tables.

A: We have converted both tables into histograms mentioned earlier and have labeled accordingly as suggested. The format has been adapted in the main text as the reviewer #2 suggested as well.

2) It may be better to list the survey questions as an appendix at the end.

A: Thank you. Please see the answer to the editor’s comment # 2. We uploaded the SI Appendix file with the original questions.

I am so impressed by the feasible solution concluded in the study. I am very glad that

you accepted this manuscript and started its reviewing process.

A: Thank you.

Reviewer #2: This is a manuscript reporting the results of a provider survey in China to assess the causes of high cesarean delivery and low labor epidural analgesia rates. The authors found that China has a high cesarean rate due to cultural and legal considerations, while the low epidural analgesia usage is impacted by lack of providers, incentives and social issues. The survey explored some methods to reduce the cesarean rate and increase the usage of epidural analgesia. While these are complex issues that, naturally, cannot be explained in a single survey, the information being reported is of interest for the health care system of the World’s largest population. The manuscript could use a little improvement in organization and language editing, as the tenses fluctuate even within a sentence.

A: Thank you.

Major critiques

Data collected via Wechat: while this is the predominant communications tool in China, can the authors estimate the penetrance of the survey – in other words, how many of each providers received the survey, not just those that opened it but did not answer.

A: Yes, there were 4,318 persons opening the survey and 32.7% (1,412/4,318) of answers or 67.3% (2906/4,318) of non-answers. It has been documented at the beginning of the result section. Additionally, we have added a geographical distribution analysis suggested by the reviewer #1.

Abstract is not fully descriptive of the results

A: We have rewritten the abstract for better descriptive wording. Thank you for pointing it out.

Please do not repeat data that exists in the tables and figures in the Results section. You should describe what the significant findings were, but the multiple numbers are confusing and require the reader to do a lot of work to comprehend.

A: Thank you. We have changed both tables into histograms for better presentation and visualization as the reviewer #1 suggested. We have changed the texts reflecting these changes along with your suggestion that only reporting the significant parts of the results with better logic.

To some extent, the survey is designed to create the result that the authors hypothesized. It is challenging to understand how the reported numbers do – or do not – support or refute the narrative. For example, “3.5-5 times as many ANs (19%) as OBs (3.8%) and MNs (5.5%) responded that the low LEA rate is related to a smaller financial risk related to CD and more risk of monetary loss for LEA (Q2-c, p < 0.001), consistent with item Q1-f, which reveals that 20% ANs, 4.0% OBs and 7.8% MNs believed that monetary gain is a possible cause for the high CD rate observed.” Does this mean that only 20% of anesthesia providers and 4% of obstetric providers believe financial incentives lead to the outcome? This seems like a small minority support the opinion being put forth. Do 80% of anesthesia and 96% of obstetric providers believe otherwise?

A: Thank you. We have rephrased this section reflecting your comments.

Minor comments

Yinao/yinbao is written three different ways throughout the document. Please use only 1 version

Introduction

1st paragraph:

Many factors likely contributed to this high CD rate, with one of the possible reasons being fear of labor pain

The fear of labor pain is independent of culture…

Low rates of labor epidural analgesia (LEA) might result in high rates

A: Thank you for pointing out. They are all unified as Yinao/yibao at the beginning of sentences or yinao/yibao if not.

“In fact, with countermeasures and education in place,” this is speculation, unless the authors have evidence they can quote. Please state the facts as known (LEA has increased in some regions) without hypothesizing why

A: We have rephrased as “given that LEA usage increased from < 1% to ≥ 31% in some hospitals or regions from 2007 to 2018” with more evidence to avoid assuming or objective statements as pointed out.

Methods

page 4: All questionnaires could be taken by either computers or smartphones based on their own choice (it’s not really a clinical practice to answer a survey)

A: We have rephrased to “The questionnaire could be taken by either computers or smartphones but was limited to one per Internet Protocol address to avoid duplicated answers from a single person or device.”

9'51'' – change to minutes & seconds

A: The changes have been made for 9 minutes and 51 seconds.

Results

Page 5: Three professionals (OBs 4% vs. ANs 20% vs. NMs 7.8%) disagreed… – this might be better to report the numbers rather than only %

A: The changes have been made across the manuscript for clarity and consistency. For example, the sentence mentioned has changed to “the 3 specialties differed (OBs 4% of 495 vs. ANs 20% of 606 vs. NMs 7.8% of 219) regarding the statement”, Thank you for your suggestion.

Discussion

Page 7: “This survey attempted to gather opinions from perinatal care providers on these issues and seek potential solutions”

…for the high rate of CD were fear of consequences…

A: The change has been made. Thank you for your suggestion.

Page 8

This "superstition culture" affects Chinese CD rate dramatically contributing up to 27% of coded “social reasons” in one study or 21% of coded “no indication” in another study. (only one study is referenced, please add both)

Obstetric anesthesiologists, the physicians play a vital role in parturient safety are also lack in China, which may have resulted in higher maternal mortality in low/middle income countries. – I don’t know what this sentence is trying to say

A: We have found a newer systematic review regarding CD rates associated with superstition culture and two other papers that address this issue outside of China. Therefore, we have rephrased accordingly to make our discussion more objective and more generalisable. The following is the updated paragraph:

“Superstitious culture surrounding the birth of the child also affects the usage of LEA and CD. Many Chinese parents/grandparents believe their child would be smarter or wealthier if the baby is delivered at a specific date and time frame, and CD is the only way to meet their wishes. This "superstitious culture" affects Chinese CD rate dramatically, with estimations of this phenomenon contributing up to 27% of coded “social reasons” in one study [17]. A total of 7 studies were collected in a systematic review reporting incidence of this phenomenon ranging from 1.1% to 19.2% [18]. Auspicious birth dates as a reason for CD has been popularized not only in China but also in Thailand and in the United States [19-21]. “

Page 9

Studies indicate that increasing incentives to providers rapidly increases LEA and decreases CD rates with better maternal and infant outcomes, while the hospital revenue increases. This might serve as a short-term solution while more AN and OB personnel are trained.13, 23 (which may repeat the American history that occurred twice.24,25 – this is not necessary)

A: The corrections are made. Thank you.

Page 10

Although modifying the national medicolegal system, billing codes…

Delete “This survey will guide NPLD-GHI in its goals, provide information on clinical practice and policy making in China and other lower to middle income countries.” That is not appropriate for a journal article, but is a statement of internal strategy

A: We have deleted “guide NPLD-GHI in its goals,” and thank you for pointing out.

Figure 1: the donut chart should be an itemized list, as the circlular format is not relevant, and the colors are not applied to the left half of the figure.

Table 1: it might be clearer if you place the percentages inside of the column (e.g. 360 (26%) )

A: Integrating the comments from both the editor and the reviewer 1, we have decided to redesign the figure for better presentation. Also, we have converted both tables into histograms as the reviewer 1 suggested.

6. PLOS authors have the option to publish the peer review history of their article ([what does this mean?](https://journals.plos.org/plosone/s/editorial-and-peer-review-process#loc-peer-review-history)). If published, this will include your full peer review and any attached files.

If you choose “no”, your identity will remain anonymous but your review may still be made public.

**Do you want your identity to be public for this peer review?** For information about this choice, including consent withdrawal, please see our [Privacy Policy](https://www.plos.org/privacy-policy).

Reviewer #1: **Yes:** Qing Ding, MD, FACOG

Reviewer #2: No

[NOTE: If reviewer comments were submitted as an attachment file, they will be attached to this email and accessible via the submission site. Please log into your account, locate the manuscript record, and check for the action link "View Attachments". If this link does not appear, there are no attachment files.]

While revising your submission, please upload your figure files to the Preflight Analysis and Conversion Engine (PACE) digital diagnostic tool, <https://pacev2.apexcovantage.com/>. PACE helps ensure that figures meet PLOS requirements. To use PACE, you must first register as a user. Registration is free. Then, login and navigate to the UPLOAD tab, where you will find detailed instructions on how to use the tool. If you encounter any issues or have any questions when using PACE, please email PLOS at figures@plos.org. Please note that Supporting Information files do not need this step.
