# Supplementary material for: Why is the labor epidural rate low and cesarean delivery rate high? A survey of Chinese perinatal care providers
Source: PLoS One. 2021 May 21;16(5):e0251345. doi: 10.1371/journal.pone.0251345 (PMC8139447; doi:10.1371/journal.pone.0251345)
Supplement: Attachment — Submitted filename: Response to Reviewers -v3.docx [file pone.0251345.s005.docx]

RE: Response to Reviewers-v3 for PONE-D-21-00405

“Why is the labor epidural rate low and cesarean delivery rate high? A survey of Chinese perinatal care providers”

Dear Dr. Wei,

Thank you and reviewers for your constructive comments and suggestions. The following is our response to your comments and reviewers’.

Thank you for your time and efforts.

Sincerely,

Ling-Qun Hu, MD

The corresponding author

=======================================

Dear Dr. Hu, 

Thank you for submitting your manuscript to PLOS ONE. After careful consideration, we feel that it has merit but does not fully meet PLOS ONE’s publication criteria as it currently stands. Therefore, we invite you to submit a revised version of the manuscript that addresses the points raised during the review process.


Please submit your revised manuscript by May 23 2021 11:59PM. If you will need more time than this to complete your revisions, please reply to this message or contact the journal office at [plosone@plos.org](mailto:plosone@plos.org). When you're ready to submit your revision, log on to <https://www.editorialmanager.com/pone/> and select the 'Submissions Needing Revision' folder to locate your manuscript file.

Please include the following items when submitting your revised manuscript:

- A rebuttal letter that responds to each point raised by the academic editor and reviewer(s). You should upload this letter as a separate file labeled 'Response to Reviewers'.
- A marked-up copy of your manuscript that highlights changes made to the original version. You should upload this as a separate file labeled 'Revised Manuscript with Track Changes'.
- An unmarked version of your revised paper without tracked changes. You should upload this as a separate file labeled 'Manuscript'.

If you would like to make changes to your financial disclosure, please include your updated statement in your cover letter. Guidelines for resubmitting your figure files are available below the reviewer comments at the end of this letter.

If applicable, we recommend that you deposit your laboratory protocols in [protocols.io](http://protocols.io/) to enhance the reproducibility of your results. Protocols.io assigns your protocol its own identifier (DOI) so that it can be cited independently in the future. For instructions see: <http://journals.plos.org/plosone/s/submission-guidelines#loc-laboratory-protocols>. Additionally, PLOS ONE offers an option for publishing peer-reviewed Lab Protocol articles, which describe protocols hosted on [protocols.io](http://protocols.io/). Read more information on sharing protocols at <https://plos.org/protocols?utm_medium=editorial-email&utm_source=authorletters&utm_campaign=protocols>.

We look forward to receiving your revised manuscript.

Kind regards,

Huafeng Wei, MD, PhD
Academic Editor
PLOS ONE

Journal Requirements:

Please review your reference list to ensure that it is complete and correct. If you have cited papers that have been retracted, please include the rationale for doing so in the manuscript text, or remove these references and replace them with relevant current references. Any changes to the reference list should be mentioned in the rebuttal letter that accompanies your revised manuscript. If you need to cite a retracted article, indicate the article’s retracted status in the References list and also include a citation and full reference for the retraction notice.

Additional Editor Comments (if provided):

Please address the comments from reviewer 2.


[Note: HTML markup is below. Please do not edit.]

Reviewers' comments:

Reviewer's Responses to Questions

**Comments to the Author**

1. If the authors have adequately addressed your comments raised in a previous round of review and you feel that this manuscript is now acceptable for publication, you may indicate that here to bypass the “Comments to the Author” section, enter your conflict of interest statement in the “Confidential to Editor” section, and submit your "Accept" recommendation.

Reviewer #1: All comments have been addressed

Reviewer #2: All comments have been addressed

2. Is the manuscript technically sound, and do the data support the conclusions?

The manuscript must describe a technically sound piece of scientific research with data that supports the conclusions. Experiments must have been conducted rigorously, with appropriate controls, replication, and sample sizes. The conclusions must be drawn appropriately based on the data presented.

Reviewer #1: Yes

Reviewer #2: Yes

3. Has the statistical analysis been performed appropriately and rigorously?

Reviewer #1: Yes

Reviewer #2: Yes

4. Have the authors made all data underlying the findings in their manuscript fully available?

The [PLOS Data policy](http://www.plosone.org/static/policies.action#sharing) requires authors to make all data underlying the findings described in their manuscript fully available without restriction, with rare exception (please refer to the Data Availability Statement in the manuscript PDF file). The data should be provided as part of the manuscript or its supporting information, or deposited to a public repository. For example, in addition to summary statistics, the data points behind means, medians and variance measures should be available. If there are restrictions on publicly sharing data—e.g. participant privacy or use of data from a third party—those must be specified.

Reviewer #1: Yes

Reviewer #2: No

A: To compliance with the Plos ONE policy to make all data underlying the findings in their manuscript fully available to the public, we have uploaded our survey findings for both Part I and Part II as S2 Appendix.

5. Is the manuscript presented in an intelligible fashion and written in standard English?

PLOS ONE does not copyedit accepted manuscripts, so the language in submitted articles must be clear, correct, and unambiguous. Any typographical or grammatical errors should be corrected at revision, so please note any specific errors here.

Reviewer #1: Yes

Reviewer #2: Yes

6. Review Comments to the Author

Please use the space provided to explain your answers to the questions above. You may also include additional comments for the author, including concerns about dual publication, research ethics, or publication ethics. (Please upload your review as an attachment if it exceeds 20,000 characters)

Reviewer #1: I have no additional comments at this time. The authors have adequately addressed your comments raised in a previous round of review and you feel that this manuscript is now acceptable for publication. Thank you for having me review the manuscript. Good luck for publishing your work. Thanks.

A: Thank you for your time and efforts.

Reviewer #2: Line 48: ‘without statistical difference among specialties.’
Also, delete: ‘It was consistent with’ and make: the most recommended solution was increasing

A: In L48-49: have added “among specialties” after “without statistical difference”, deleted “It was consistent with” and reconstructed it as “The most recommended solution was increasing the anesthesia workforce…” in the sentence after.

Line 50: This is a confusing sentence, as it is not clear which group relates to the numbers. Please revise to something like: However, the top solution provided by the two non-anesthesia specialties was different from the one proposed by anesthesiologists. The later (83%, 504/606) suggested increasing the incentive to provide the service is more effective.

A: Thank you for your suggestion. It has been changed accordingly, it is located at L51-52, now.

Line 95: there are two commas

A: One extra comma is deleted. Thank you.

Line 124: percentages, please

A: Two percentages have been added as “either Android (65.4%, 923/1412) or iOS devices (33.9%, 478/1412)”, now located in L123.

Line 261: fewer complications

A: In L260, “less complications” has been replaced by “fewer complications”.

Line 263: Top solution (top one is redundant)

A: “one” has been deleted accordingly in L262.

Line 268: however, the specialty does not officially exist (I am assuming the people officially exist!)

A: Yes, the people officially exist and some of them deliver babies. What a catch! Thank you. The new phrase is “however, the specialty does not officially exist in China.” at L267-268.

Line 286: delete ‘seriously’

A: ‘seriously’ has been deleted in L285. Thank you.

Line 295: increasingly popular (‘more’ is redundant)

A: ‘more’ has been deleted in L294. Thank you.

7. PLOS authors have the option to publish the peer review history of their article ([what does this mean?](https://journals.plos.org/plosone/s/editorial-and-peer-review-process#loc-peer-review-history)). If published, this will include your full peer review and any attached files.


If you choose “no”, your identity will remain anonymous but your review may still be made public.


**Do you want your identity to be public for this peer review?** For information about this choice, including consent withdrawal, please see our [Privacy Policy](https://www.plos.org/privacy-policy).

Reviewer #1: No

Reviewer #2: No

Thank you, both the editors and the reviewers.
